# Association of Matrix Metalloproteinases -7, -8 and -9 and TIMP -1 with Disease Severity in Acute Pancreatitis. A Cohort Study

**DOI:** 10.1371/journal.pone.0161480

**Published:** 2016-08-25

**Authors:** Eija Nukarinen, Outi Lindström, Krista Kuuliala, Leena Kylänpää, Ville Pettilä, Pauli Puolakkainen, Antti Kuuliala, Mari Hämäläinen, Eeva Moilanen, Heikki Repo, Johanna Hästbacka

**Affiliations:** 1 Department of Perioperative, Intensive Care and Pain Medicine, University of Helsinki and Helsinki University Hospital, Helsinki, Finland; 2 Department of GI Surgery, University of Helsinki and Helsinki University Hospital, Helsinki, Finland; 3 Department of Bacteriology and Immunology, University of Helsinki and Helsinki University Hospital, Helsinki, Finland; 4 The Immunopharmacology Research Group, University of Tampere School of Medicine and Tampere University Hospital, Tampere, Finland; Van Andel Institute, UNITED STATES

## Abstract

**Objectives:**

Several biomarkers for early detection of severe acute pancreatitis (SAP) have been presented. Matrix metalloproteinases (MMP) and their tissue inhibitors (TIMP) are released early in inflammation. We aimed to assess levels of MMP-7, -8, -9 and TIMP-1 in acute pancreatitis (AP) and explore their ability to detect disease severity. Our second aim was to find an association between MMPs, TIMP and creatinine.

**Methods:**

We collected plasma samples for MMP-7, -8, -9 and TIMP-1 analyses from 176 patients presenting within 96 h from onset of acute pancreatitis (AP) symptoms. We used samples from 32 control subjects as comparison. The revised Atlanta Classification was utilised to assess severity of disease. Receiver operating characteristic curve analysis and Spearman´s *Rho*-test were utilised for statistical calculations.

**Results:**

Compared with controls, patients showed higher levels of all studied markers. MMP-8 was higher in moderately severe AP than in mild AP (p = 0.005) and MMP-8, -9 and TIMP-1 were higher in severe than in mild AP (*p*<0.001, *p* = 0.005 and *p* = 0.019). MMP-8 detected SAP with an AUC of 0.939 [95% CI 0.894–0.984], LR+ 9.03 [5.30–15.39]. MMP-8, -9 and TIMP-1 failed to discern moderately severe AP from SAP. MMP-7 was not different between patient groups. MMP-7 and TIMP-1 correlated weakly with creatinine (*Rho* = 0.221 and 0.243). MMP-8 might be a useful biomarker in early detection of SAP.

## Introduction

Acute pancreatitis (AP), classified as mild, moderately severe or severe by the revised Atlanta Classification (RAC) system [1], is a potentially life-threatening condition causing a great burden to both the patient and the healthcare system. Although mainly presenting in its mild form with a recovery time of a few days, approximately 20% of cases develop severe acute pancreatitis (SAP) requiring long hospital stay and admission to the intensive care unit (ICU) and comprising a mortality of 15–20% [2]. The mortality is primarily due to multi-organ failure and pancreatic necrosis infection [3]. The histological findings of experimental pancreatitis include cell necrosis and haemorrhage. Furthermore, an activation of trypsinogen to trypsin together with neutrophil infiltration occurs [4].

Patients with SAP should be admitted early to the ICU [1]. Several biochemical markers have been studied with the aim to discern severe cases early in the disease course. Serum amylase [5] together with C-reactive protein (CRP), procalcitonin (PCT), interleukins 6 and -8, trypsinogen and calcium are examples of laboratory markers reported to detect SAP [6]. Elevation of CRP is slow in the early phase of AP and not specific for the disease. Some studies have shown PCT to be a good predictor of severity but the results are still conflicting. Despite their limitations CRP and PCT are currently among the most commonly used laboratory markers in this respect [7,8]. However, a standardised method for severity assessment is lacking [8]. A laboratory marker with high specificity and sensitivity for the detection of severity early in AP would be desirable in order to commence timely appropriate treatment and by that avoid preventable complications in patients likely to develop SAP [9]. Moreover, the measurement method should preferably be simple and rapid to perform.

Matrix metalloproteinases (MMPs) are a group of enzymes involved in processes such as inflammation, degradation and turnover of the extracellular matrix as well as angiogenesis and tumour growth. Activated MMPs are tightly regulated by naturally occurring α-macroglobulines and tissue inhibitors of matrix metalloproteinases (TIMPs) [10]. TIMPs-1, -2,-3 and -4 all inhibit MMPs, albeit non-specifically [11]. Of these, TIMP-1 is one of the most important regulators of MMP-8 and -9 [12]. MMP-7 expression occurs in the epithelia of non-inflamed exocrine glands and mucosal epithelium and is reported to be upregulated after bacterial exposure [10, 13]. Moreover, MMP-7 is found in chronic pancreatitis and in pancreatic carcinoma [14]. Secreted from neutrophils, MMP-8 plays a diverse role in both acute and chronic inflammation [12] by enhancing release and causing proteolytic modification of leukocyte migration navigating chemokines [15]. MMP-8 as a predictor of disease severity and progression has been studied in several inflammatory conditions [16]. The role of MMP-7 and -8 in the context of AP is, however, not well determined. Experimentally, MMP-8 on the surface of polymorphonuclear (PMN) cells is found to exhibit protease activity towards serine protease inhibitors in inflammation. Activation of the serine protease inhibitors could theoretically increase the activity of serine proteases such as trypsin and chymotrypsin [17]. The role of MMP-9 and TIMP-1 have been extensively studied in AP and serum levels of MMP-9 have been found to be of possible prognostic significance [4, 18, 19, 20, 21]. In rodent AP models the pancreatic enzyme trypsin caused remarkable MMP-9 release [22], whereas inhibition of MMP-9 reduced trypsinogen activation [4].

In SAP acute kidney injury (AKI) is one of the key manifestations of organ dysfunction. An elevation of serum creatinine has been used as a predictor of disease severity [23] and occurrence of pancreatic necrosis [24] as well as death [25]. The concentration of MMP-8 in serum has been shown to be elevated in septic children with AKI compared to those without AKI [26], but less is known about the relationship between MMP-8 and AKI in AP. In a recent rodent study on experimental SAP, MMP-9 was found to be markedly elevated in the kidney within 12 h from the induction of pancreatitis preceding a later rise in serum creatinine [27]. In humans the association of MMPs to organ failure in patients with AP is poorly investigated. Additionally, the data on the role of MMP-7 and -8 in AP in humans is scarce.

We hypothesized that elevated levels of plasma MMP -7, -8, -9 or TIMP-1 can be found early in AP and that these elevated levels may detect the degree of disease severity. We therefore aimed to assess the association of plasma concentration of MMP-7, -8, -9 and TIMP-1 within 96 h from the onset of symptoms with the severity of AP as classified by the RAC and to compare these concentrations to those of control subjects. Our second aim was to study the association between early MMP -7, -8, -9 and TIMP-1 concentrations and plasma creatinine concentration.

## Patients and Methods

Our study is a single centre cohort study. We collected laboratory samples from two study cohorts at Helsinki University Hospital, which is a tertiary level centre, during a time period from March 2011 to August 2014. Each of the studies, together with the use of blood samples from adult healthy voluntary subjects, was granted approval by the Ethical Committee of the Department of Surgery at Helsinki University Hospital. We conducted the study according to the principles of the World Medical Association´s Declaration of Helsinki. For our study we analysed plasma samples from 176 non-consecutive patients presenting with AP within 96 h from onset of symptoms. Of these, 11 patients participated in the earlier published FINNAKI study focusing on ICU treated AKI [28]. There are no published studies on the variation of MMP-7, -8 or TIMP-1-levels in AP, hence we did not perform a power analysis to calculate sample size. The patients or their next-of-kin gave their written informed consent to our study. We obtained control samples from healthy healthcare personnel volunteering to serve as control subjects after a verbal informed consent, as approved by the Ethics Committee. Consent from control subjects, including date of consent and identification data, were recorded in writing separately.

We included patients over 18 years of age with AP. The diagnosis of AP was based on typical clinical findings including epigastric pain, nausea and vomiting, an elevated plasma amylase concentration of at least 3-fold the upper reference limit and/or typical radiological appearance of AP on computed tomography (CT). We categorised the severity of AP in our study patients retrospectively during the hospital stay according to the RAC system [1] as mild (Class 0), moderately severe (Class 1; local or systemic complication or transient organ failure [OF]) or severe (Class 2; persistent OF). We defined renal, circulatory or respiratory OF by the modified Marshall score [29]. We calculated Acute Physiology and Chronic Health Evaluation (APACHE) II scores on admission to the hospital and recorded the first SOFA score for patients admitted to the ICU. Furthermore, we recorded the presence of chronic cardiovascular, renal or lung diseases together with a suspected or diagnosed sepsis at admission. Within 96 h from the onset of AP symptoms we drew blood samples in 9 ml EDTA (ethylene diamine tetra acetic acid) plasma tubes from the included patients. For comparison we used blood samples from adult healthy voluntary control subjects (n = 32). After centrifugation, we stored the samples at -70°C until analysis. We measured MMP-7, MMP-8, MMP-9 and TIMP-1 levels in plasma by enzyme-linked immunoassay (ELISA). The used reagents were manufactured by R&D Systems Europe Ltd (Abingdon, UK) and the assay protocol and cross-reactivity of the antibodies are described in the following datasheets:

https://resources.rndsystems.com/pdfs/datasheets/dy907.pdf (MMP-7) https://resources.rndsystems.com/pdfs/datasheets/dy908.pdf (MMP-8) https://resources.rndsystems.com/pdfs/datasheets/dy911.pdf (MMP-9)
https://resources.rndsystems.com/pdfs/datasheets/dy970.pdf (TIMP-1)

We measured absorbance with Victor3 Multilabel Counter (Perkin Elmer, Finland) and calculated the results against standard curve using smoothed spline method by MultiCalcTM (Perkin Elmer, Finland). We calculated inter-assay coefficient of variation for each analyte by measuring a standard sample at the same location of the plate in every plate included in the analysis. The detection limits and inter-assay coefficients of variation were 7.8 pg/ml and 4.1% for MMP-7, 15.6 pg/ml and 2.5% for MMP-8, 7.8 pg/ml and 4.9% for MMP-9 and 7.8 pg/ml and 3.1% for TIMP-1. We recorded the first plasma creatinine, CRP and amylase values (reference range 50–100 μmol/l, < 3 mg/l and 25–120 IU/l, respectively) measured in hospital and the presence of AKI at admission.

We compared categorical variables using Fisher´s exact test and non-parametric variables between two or several groups using Mann-Whitney and Kruskall-Wallis non-parametric tests, respectively. Correlations between non-parametric variables were calculated using Spearman´s *Rho*-test. To test the diagnostic ability of the variables in detecting the severity of disease we used ROC analysis and calculated areas under the curve (AUC) with 95% confidence intervals (95% CI). We used the Youden method to determine the optimal cut-off values. For the calculated cut-off values we calculated sensitivity, specificity, and the positive likelihood ratio (LR+) with 95% CI. A *p*-value of < 0.05 was used by us to designate statistical significance. We performed all statistical analyses using SPSS 20.0 (IBM, Chicago, IL).

## Results

Of the 178 patients recruited, a total of 176 patients were included in our study. Samples from two patients were lost for analysis. 128 patients with AP were classified as mild, 25 as moderately severe and 23 as SAP according to the RAC. All patients with SAP were treated in the ICU. None of the patients were diagnosed with sepsis at inclusion to the study. The demographic and clinical variables of our patients are shown in Table 1. Two patients classified as having SAP died in the ICU.

**Table 1:** Patient demographics and clinical variables.

	Mild	Moderately severe	Severe	*p*-value
	(n = 128)	(n = 25)	(n = 23)	
**Age**	**51 (22–86)**	**54 (38–91)**	**48 (26–82)**	***ns***
**Gender male (%)**	**90 (70.3)**	**17 (68)**	**15 (65.2)**	
**Comorbidities**				
**Cardiovascular (%)**	**48 (37.5)**	**13 (52)**	**6 (26.1)**	
**Chronic lung (%)**	**15 (11.7)**	**2 (8)**	**2 (8.7)**	
**Renal (%)**	**3 (2.3)**	**2 (8)**	**0 (0)**	
**Aetiology**				
**Alcohol (%)**	**80 (62.5)**	**14 (56)**	**17 (74)**	
**Gallstone (%)**	**24 (18.8)**	**9 (36)**	**4 (17)**	
**Other/unknown (%)**	**24 (18.8)**	**2 (8)**	**2 (9)**	
**APACHE II**	**5 [3–7]**	**6 [5–9]**	**11 [6–18]**	**<0.001**
**SOFA**	**N/A**	**N/A**		
**ICU admission (n)**	**0**	**0**	**23**	
**Days of hospitalization**	**4 [3–6]**	**10 [6–13]**	**15 [12–27]**	
**Hospital mortality**	**0**	**0**	**2**	
**Organ failure**				
**Circulatory (%)**	**0 (0)**	**2 (8)**	**12 (52)**	
**Respiratory (%)**	**0 (0)**	**8 (32)**	**20 (87)**	
**Renal (%)**	**0 (0)**	**0 (0)**	**15 (65)**	
**Mechanical ventilation**	**0**	**0**	**10**	
**RRT**	**0**	**0**	**7**	
**Sepsis**	**0**	**0**	**0**	
**CRP (mg/l)**	**39 [10–115]**	**36 [14–221]**	**75 [20–189]**	***ns***
**Creatinine (μmol/l)**	**62 [54–79]**	**65 [49–89]**	**70 [52–107]**	***ns***
**Amylase (IU/l)**	**295 [135–722]**	**460 [186–1079]**	**481 [163–1162]**	***ns***

Severity of pancreatitis classified by the revised Atlanta classification system. Age in years expressed as mean (range). Categorical variables are expressed as numbers. Continuous variables are expressed as median and interquartile range [IQR]. APACHE; Acute physiology and Chronic Health Evaluation Score. SOFA; Sepsis-related Organ Failure Assessment. RRT; renal replacement therapy. CRP; C-reactive protein. Creatinine; plasma concentration of creatinine. Amylase; plasma concentration of amylase. P denotes statistical significance at the level of 0.05.

**Fig 1:**
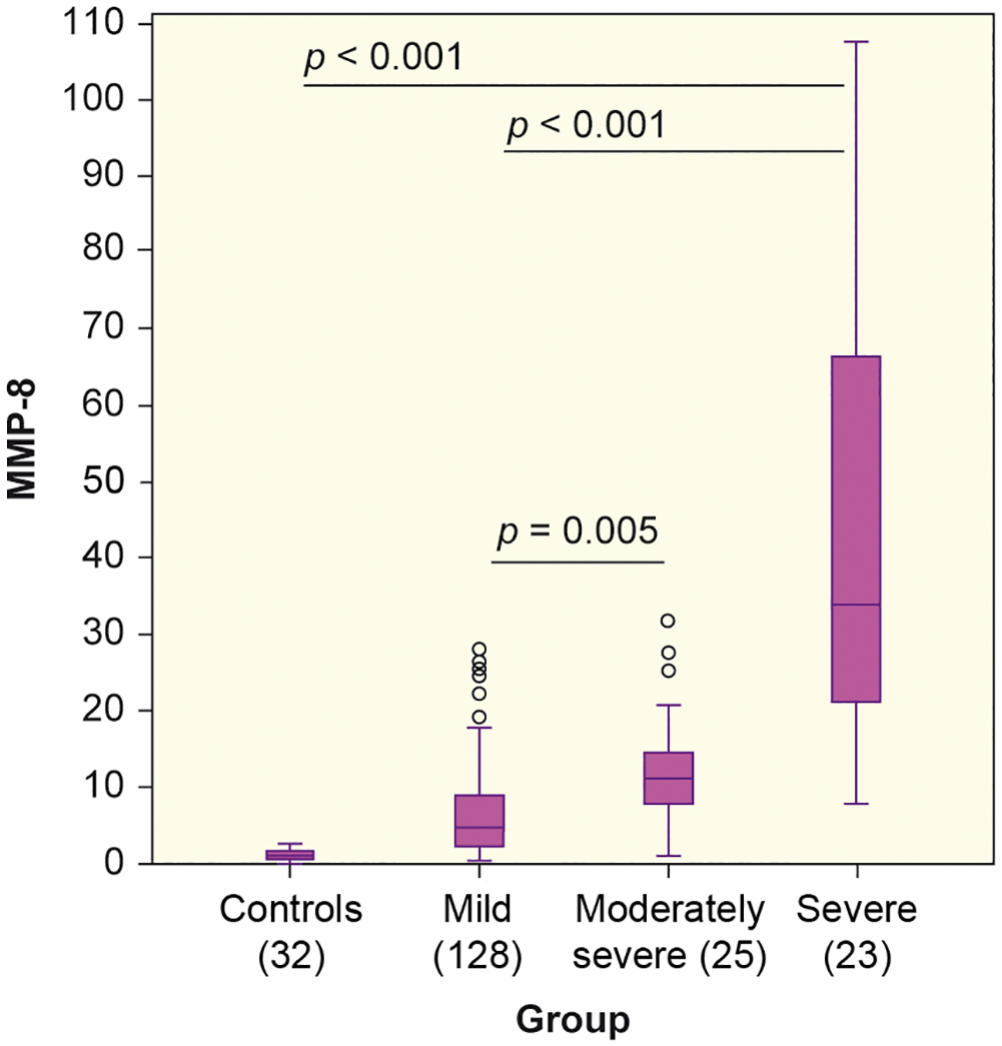
Levels of matrix metalloproteinase -8 (MMP-8) in patient groups and controls. Y-axis: MMP-8 in ng/ml. X-axis: Control subjects and patients classified by the revised Atlanta classification system.

**Fig 2:**
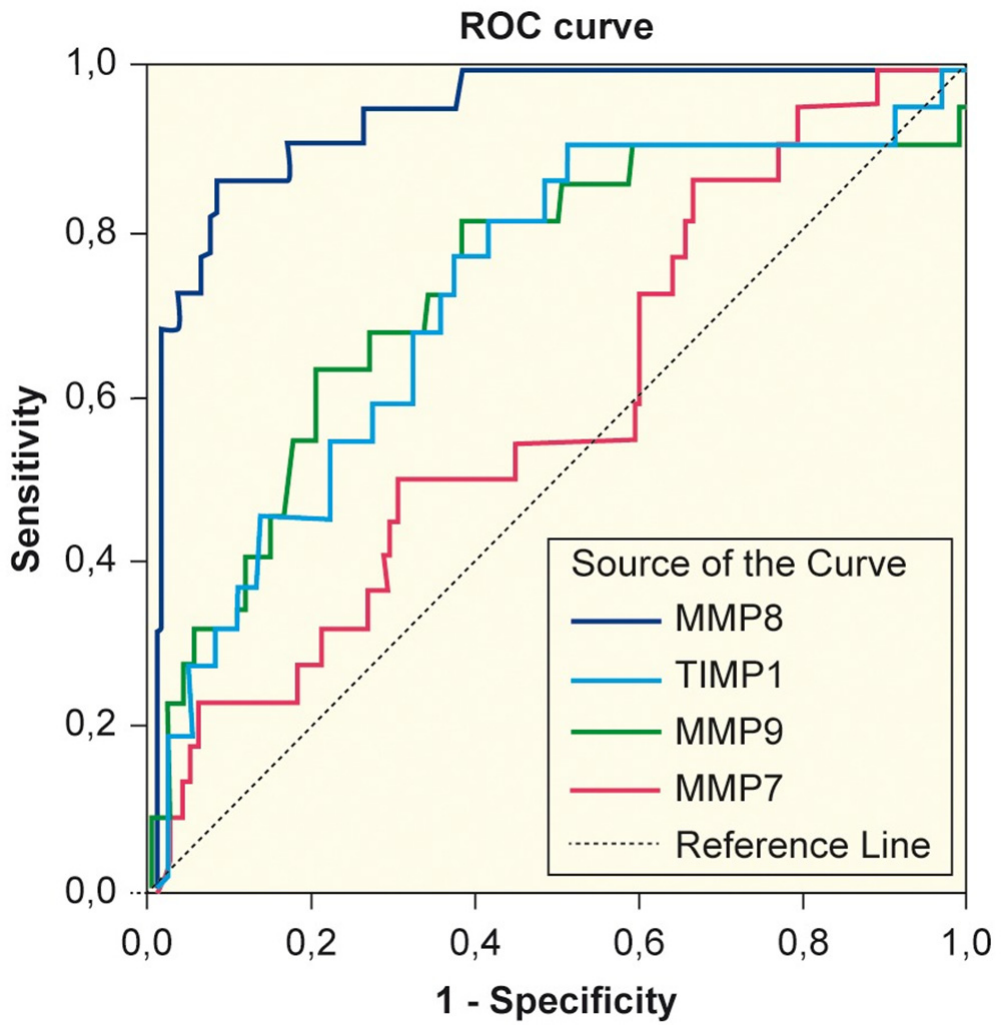
Receiver operating curves (ROC) to test detection of severe acute pancreatitis. ROC curves demonstrating the ability of MMP-7, MMP-8, MMP-9 and TIMP-1 to detect severe acute pancreatitis. MMP; matrix metalloproteinase. TIMP; tissue inhibitor of matrix metalloproteinase.

The median concentrations of MMPs and TIMP-1 in patients and controls are presented in Table 2. Compared with controls, patients showed significantly higher concentrations of all studied markers (*p*<0.001). The distribution of MMP-8 with respective *p*-values between groups is depicted in Fig 1. The concentration of MMP-8 was able to detect SAP with an AUC of 0.939 [95% CI 0.894–0.984] (Fig 2). The optimal cut-off value of MMP-8 for correct classification was 16.53 ng/ml. At this cut-off point, the sensitivity and specificity were 83% and 91%, respectively. The AUCs, cut-off values with sensitivity, specificity and LR+ for MMP-8,-9 and TIMP-1 are shown in Table 3. MMP-9 showed no concentration difference between mild and moderately severe AP (*p* = 0.069) or between moderately severe AP and SAP (*p* = 1.000), but it discerned mild AP from SAP (*p* = 0.005). There was no difference in MMP-7 concentration between any of the patient groups (*p* = 1.000). Neither did MMP-7 detect SAP, the calculated AUC being 0.590 [95%CI 0.465–0.714]. The concentrations of CRP and amylase did not differ significantly between patient groups (Table 1). There was a statistically significant (*p*<0.001) but weak positive correlation (Rho = 0.35) between MMP-8 and CRP. S1 Fig depicts the receiver operating curves for the ability of CRP, amylase and APACHE II to detect SAP.

**Table 2:** Levels of matrix metalloproteinase (MMP) -7,-8,-9 and tissue inhibitor of matrix metalloproteinase-1 (TIMP-1) in acute pancreatitis (AP) patients and controls.

	Controls	Mild	Moderately severe	Severe	*p*-value
**MMP-7 (ng/ml)**	**0.18 [0.08–0.30]**	**0.68 [0.28–1.17]**	**0.53 [0.33–1.13]**	**0.88 [0.39–1.49]**	**<0.001**
**MMP-8 (ng/ml)**	**0.88 [0.66–1.54]**	**4.80 [2.26–9.01]**	**11.19 [7.48–14.58]**	**33.84 [19.44–73.34]**	**<0.001**
**MMP-9 (ng/ml)**	**39.60 [26.30–50.13]**	**135.77 [60.70–266.90]**	**317.26 [61.93–120.23]**	**314.59 [233.40–486.31]**	**<0.001**
**TIMP-1 (ng/ml)**	**81.22 [70.50–91.47]**	**146.80 [105.54–240.1]**	**150.68 [114.42–308.74]**	**302.58 [173.93–526.04]**	**<0.001**

Continuous variables are expressed as median and interquartile range [IQR]. Groups defined by severity of disease according to the revised Atlanta classification system. A p-value < 0.05 denotes statistical significance in comparison to controls.

**Table 3:** Statistical characteristics of MMP -8, -9 and TIMP-1 describing their ability to detect severe acute pancreatitis.

Variable	AUC [95% CI]	Cut-off value	Sensitivity [95% CI]	Specificity [95% CI]	LR+ [95% CI]
**MMP-8**	**0.939 [0.894–0.984]**	**16.53**	**0.83 [0.67–0.98]**	**0.91 [0.86–0.95]**	**9.3 [5.30–15.39]**
**MMP-9**	**0.722 [0.606–0.838]**	**230.25**	**0.78 [0.61–0.95]**	**0.63 [0.55–0.70]**	**2.10 [1.56–2.83]**
**TIMP-1**	**0.737 [0.614–0.860]**	**265.55**	**0.61 [0.41–0.81]**	**0.80 [0.73–0.86]**	**3.00 [1.91–4.73]**

MMP; matrix metalloproteinase. TIMP; tissue inhibitor of matrix metalloproteinase. AUC; Area under the curve for the studied variable to predict SAP. CI; confidence interval. LR+; positive likelihood ratio.

Concerning TIMP-1, a difference in concentration between mild AP and SAP was noted (*p* = 0.019), whereas it failed to show any concentration difference between mild AP and moderately severe AP (*p* = 1.000) or moderately severe and severe disease (*p* = 0.650).

There was no difference in plasma creatinine concentrations between patients grouped by severity of disease (Table 1). MMP-7 and TIMP-1 correlated weakly (Rho = 0.221, *p* = 0.003 and Rho = 0.243, *p* = 0.001) with creatinine. No correlation was, however, found between plasma creatinine and MMP-8 or -9.

## Discussion

In our study we found elevated concentrations of MMP-7, -8, -9 and TIMP-1 in plasma of AP patients within 96 h from the onset of symptoms compared with healthy controls. The level of MMP-8 was significantly higher in SAP than in mild AP. A MMP -8 level exceeding 16.53 ng/ml (LR+ 9.03 [95% CI 5.30–15.39]) performed well in detecting SAP with a good level of sensitivity and specificity. Neither MMP-8, -9 nor TIMP-1 were able to discern moderately severe AP from SAP.

The role of MMP-8 in SAP is to date not well determined. MMP-8 and -9 are both known to be released early in inflammation [30]. The initial inflammatory reaction in SAP resembles the one seen in septic shock but without an infectious cause [31]. Autodigestive processes lead to neutrophil and macrophage infiltration into the pancreas, which in severe cases can cause sepsis due to increased bacterial translocation from the gut to the circulation [3]. In experimental SAP MMP-9 has been shown to be involved in PMN cell migration and bacterial translocation in the intestine [20]. MMP-8 participates in inflammatory processes both by activation and inactivation of PMN cell attracting chemokines [15]. In severe sepsis and septic shock, MMP-8 released from neutrophil granulocytes has been reported to associate with an unfavourable outcome [32]. Because of the sepsis- induced considerable rise in MMP-8 concentration [33] we assessed the presence of sepsis at study admission without positive findings. The elevation of MMP-8 in our study could therefore be a consequence of a non-infectious, inflammatory release from PMN cells due to AP, as explained above. The levels of MMP-8 in patients in our study were lower compared with those found in sepsis patients [32]. It is possible that the inflammatory reaction in our AP patients was less pronounced at the time of sampling than in the study of *Lauhio et al* [32], or that the time point of sampling from onset of the reaction was different. To our knowledge, the association of MMP-8 with the severity of AP has not been reported earlier.

Being non-specific for AP, MMP-8 should not be used as a marker for finding AP in patients but could be utilised in discerning disease severity. The inability of MMP-8 in discerning moderately severe AP from SAP could be explained by the small number of patients with SAP in our study. Nevertheless, in our study MMP-8 performed considerably better than CRP or P- amylase in detecting SAP. Compared with currently used laboratory markers MMP-8 could be advantageous in discerning SAP from mild AP due to its early release in inflammation [34]. The AUC, sensitivity and specificity and LR+ of MMP-8 for predicting SAP in our study were relatively high and comparable to those obtained by a meta-analysis, in which PCT was found to be a good marker for diagnosing severity of early AP with an AUC of 0.94, sensitivity 89% and specificity of 84% [35].

In accordance to our findings, an elevated concentration of MMP-9 in patients with SAP compared to controls has been detected at admission [18] and during ICU stay [19]. Moreover, in our study MMP-9 was able to detect SAP with a reasonable AUC, albeit inferior to the performance of MMP-8 (Table 3).

MMP-7 is reported to be expressed in the epithelia of gastrointestinal ulcers [36] and elevated levels of serum MMP-7 in pancreatic carcinoma patients is associated to decreased survival [37]. Importantly, in inflammation MMP-7 activates tumour necrosis factor (TNF) and promotes neutrophil influx by creation of chemokine gradients [10]. Nevertheless, in our study MMP-7 in plasma did not perform well in detecting AP in any of the patient groups, alluding MMP-7 to primarily be an on-site effector molecule.

TIMP-1 inhibits MMP-9 in a 1:1 fashion [11]. The role of TIMP-1 as a marker of severity of AP has not been thoroughly investigated. In a small study by Wereszyczynska-Siemiatkowska et al an imbalance of measured levels of MMP-9 and TIMP-1 was found, suggesting failure of endogenous TIMP-1 to prevent excessive MMP-9-activation in AP [19]. In our study TIMP-1 predicted SAP with an AUC of 0.737 which is close to the AUC of MMP-9 (0.722).

In a recent rodent study on experimental SAP MMP-9 was found to be substantially elevated in the kidney within 12 h of the induction of pancreatitis preceding a later rise in serum creatinine [27]. We assessed MMP-9 in plasma, but found no correlation with plasma creatinine. Neither did we found any correlation between MMP-8 and plasma creatinine concentration. Concerning MMP-7 and TIMP-1, we found a weak correlation to plasma creatinine. *Surendran et al* detected MMP-7 in the epithelial cells of murine kidneys exposed to experimental renal injury, but not in inflammatory cells, suggesting a reparative function for the enzyme [38]. In a recently published study conducted on 53 abdominal surgery patients with sepsis, TIMP-1 appeared to be a possible diagnostic biomarker for sepsis-associated AKI [39]. However, our results do not provide sufficient evidence to advocate its use in AP.

This is to the best of our knowledge the first study to assess the association between MMP-7, -8 and TIMP-1 and severity of AP. Moreover, the relationship of plasma creatinine to MMPs in AP patients has not been reported earlier. However, there are well acknowledged limitations in our study. First, the number of SAP patients in our study is small enabling the occurrence of a type I error in the results. Our results should therefore be validated in a larger study. Second, the timespan allowed from onset of symptoms to sampling was relatively long considering the early release of MMPs in acute inflammation [34] and therefore our results may reflect different stages of the developing disease. However, the time to sampling in our study reflects clinical reality. Third, despite a notable elevation of MMP-8 in AP in our study the pathophysiologic mechanism to clarify its exact role is still lacking and it is possible that our findings reflect inflammation in general rather than a specific response to AP development. This needs to be further investigated. Fourth, the ELISA-method used for the analysis of MMP-8 in our study is currently unsuitable for use in an emergency situation. A rapid assay method, such as used in dentistry, for the detection of elevated MMP-8 [40] could be useful for this purpose. Fifth, using plasma creatinine as a surrogate for AKI instead of combining it with urine output [41] may be considered insufficient. Creatinine, together with urea concentration is, however, still widely used for this purpose [42].

## Conclusion

We conclude that plasma concentrations of MMP-7, MMP-8, -9 and TIMP-1 within 96 h from the onset of AP symptoms are elevated in AP patients compared with healthy controls. SAP may be detected with a high degree of sensitivity and specificity by measuring plasma MMP -8. MMP-8 may also discern mild from moderately severe AP. Used as a marker for AKI in our study, creatinine correlated poorly with MMP-7, -8, -9 and TIMP-1.

## Supporting Information

S1 Dataset Data collected from study patients. (SAV)

S1 Fig ROC curve depicting the ability of MMP-8, CRP, APACHE II and amylase to detect SAP. (TIF)
