# Supplementary figures and images for: Association of Matrix Metalloproteinases -7, -8 and -9 and TIMP -1 with Disease Severity in Acute Pancreatitis. A Cohort Study
Source: PLoS One. 2016 Aug 25;11(8):e0161480. doi: 10.1371/journal.pone.0161480 (PMC4999158; doi:10.1371/journal.pone.0161480)

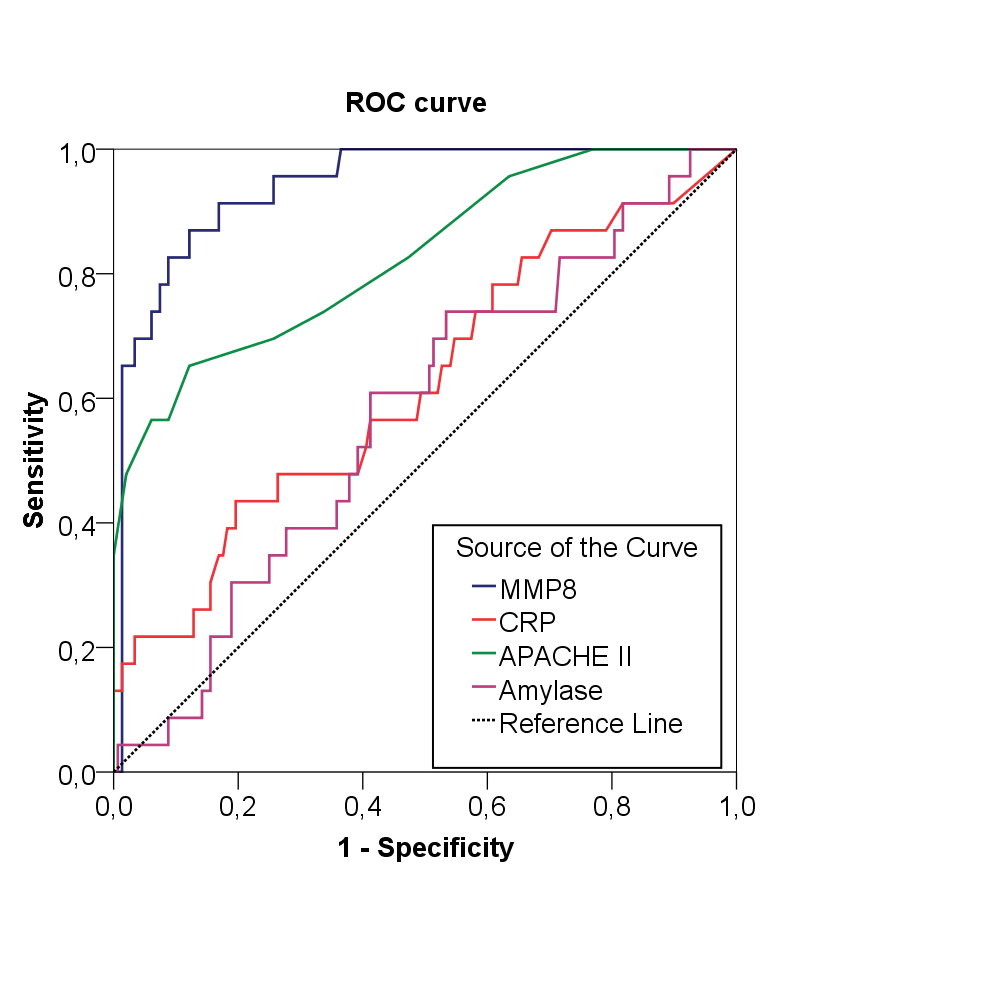

Supplement: S1 Fig — (TIF) [file pone.0161480.s002.tif]
